# Direct ethanol production from starch using a natural isolate, *Scheffersomyces shehatae*: Toward consolidated bioprocessing

**DOI:** 10.1038/srep09593

**Published:** 2015-04-22

**Authors:** Ayumi Tanimura, Minako Kikukawa, Shino Yamaguchi, Shigenobu Kishino, Jun Ogawa, Jun Shima

**Affiliations:** 1 Division of Applied Life Sciences, Graduate School of Agriculture, Kyoto University, Kitashirakawa Oiwake-cho, Sakyo-ku, Kyoto 606-8502, Japan; 2 Research Division of Microbial Sciences, Kyoto University, Kitashirakawa Oiwake-cho, Sakyo-ku, Kyoto 606-8502, Japan; 3 Faculty of Law, Ryukoku University, 67 Fukakusatsukamoto-cho, Fushimi-ku, Kyoto 612-5662, Japan

## Abstract

Consolidated bioprocessing (CBP), which integrates enzyme production, saccharification and fermentation into a one-step process, is a promising strategy for cost-effective ethanol production from starchy biomass. To gain insights into starch-based ethanol production using CBP, an extensive screening was undertaken to identify naturally occurring yeasts that produce ethanol without the addition of any amylases. Three yeast strains were capable of producing a significant amount of ethanol. Quantitative assays revealed that *Scheffersomyces shehatae* JCM 18690 was the strain showing the highest ethanol production ability. This strain was able to utilize starch directly, and the ethanol concentration reached 9.21 g/L. We attribute the ethanol-producing ability of this strain to the high levels of glucoamylase activity, fermentation potential and ethanol stress tolerance. This study strongly suggests the possibility of starch-based ethanol production by consolidated bioprocessing using natural yeasts such as *S. shehatae* JCM 18690.

Increasing energy costs and energy shortages in recent years have become problems of global significance. These factors, along with mounting evidence of climate change and its effects, have prompted a search for new sources of renewable energy. Starchy biomass is a cheap and abundant renewable carbon source in bioethanol production^1^,^2^. In addition, the accumulation of excess starchy wastes can in itself cause problems; for example, vast amounts of cassava and potato pulp accumulate in Thailand and China, respectively^3^,^4^. Therefore, it may be important to develop cost-effective ethanol production processes with a suitable energy balance in order to foster the development of a low-carbon society.

At present, the cost associated with supplying the large quantities of enzymes required to produce bioethanol makes it a less competitive fuel^1^,^5^,^6^. Eliminating the need for saccharifying enzymes in bioethanol production is thus considered a key step toward reducing its cost^6^,^7^,^8^. The single-step process enable a potential for chemical cost saving as well as a significant reduction in operating cost. Consolidated bioprocessing (CBP), which integrates enzyme production, saccharification and fermentation into a one-step process, is a promising strategy for cost-effective ethanol production from starchy biomass.

It has been well established that bioethanol can be produced by yeasts, such as *Saccharomyces cerevisiae*, *Scheffersomyces stipitis*, and *Kluyveromyces marxianus*^9^,^10^,^11^. These strains assimilate glucose and other monosaccharides derived from enzymatic hydrolysis of starch. However, there have been few reports of natural yeast strains that can yield an amylolytic enzyme and simultaneously produce ethanol from starch^12^,^13^. Although researchers have attempted to construct genetically engineered yeasts which express amylolytic enzymes^1^,^6^, the use of such yeasts is associated with regulations^14^, and these increase production costs in two main ways. First, the regulations require that the genetically engineered yeasts be physically contained in order to prevent their escaping into the environment. A special enclosure is thus required to confine them, as well as a sterilization system. Second, biological containment is also required in order to limit the survival and spread of the yeasts in the environment by means of genetic control^15^.

In this study, to gain insights into ethanol production from starch through CBP, a comprehensive screening was performed for natural yeast strains that could produce high levels of ethanol from starch by a one-step CBP conversion without the need for addition of enzymes. We also provide the possible mechanisms for the direct production of ethanol from starch.

## Results

### Extensive screening of the starch-assimilating ability of yeasts

In a primary screening, we examined the starch-assimilating ability of yeast strains isolated from soil samples in the Kyoto area of Japan. Of the 530 yeast strains tested, approximately 79% (total 419 strains) grew on the medium, and these strains were used for secondary screening.

### Evaluation of the ethanol-producing ability of selected yeasts

The 419 starch-assimilating strains were cultivated in 10% starch liquid medium for 6 days, and the ethanol concentration was determined under aerobic and anaerobic conditions. Under aerobic conditions, no strain produced a significant amount of ethanol. Under anaerobic conditions, however, 3 strains (*S. shehatae* JCM 18690, ATY945 and ATY1112) were found to produce more than 6 g/L (overall ethanol yield; g ethanol per liter of culture) ethanol, with *S. shehatae* JCM 18690 producing the greatest amount. The amount of ethanol produced by *S. shehatae* JCM 18690 reached 9.78 g/L under the experimental conditions employed in secondary screening. We previously reported that *S. shehatae* JCM 18690 is a thermotolerant xylose-fermenting strain^16^. The other strains were not taxonomically identified.

### Tentative identification of ATY945 and ATY1112

To taxonomically identify strains ATY945 and ATY1112, 26S rDNA sequencing was performed. The sequences of the D1/D2 domain of the 26S rDNA of strains ATY945 and ATY1112 were deposited in the DNA Data Bank of Japan (DDBJ) under accession nos. AB985632 and AB985633, respectively. The 26S rDNA sequences of strains ATY945 and ATY1112 showed 99% identity to that of *Candida subhashii* strain UAMH 10744 (EU836708) and 99% identity to that of *Scheffersomyces* sp. BG090809.6.7.3.1.9 (JN805246), respectively. Therefore, we regard strains ATY945 and ATY1112 as belonging to *Candida subhashii* and *Scheffersomyces* sp., respectively.

### Ethanol production from starch of the screened strains

The fermentation profiles of the screened strains were examined. We employed *S. shehatae* NBRC 1983 as a control strain, because this strain is known to be a starch-assimilating strain.

**Figure 1:**
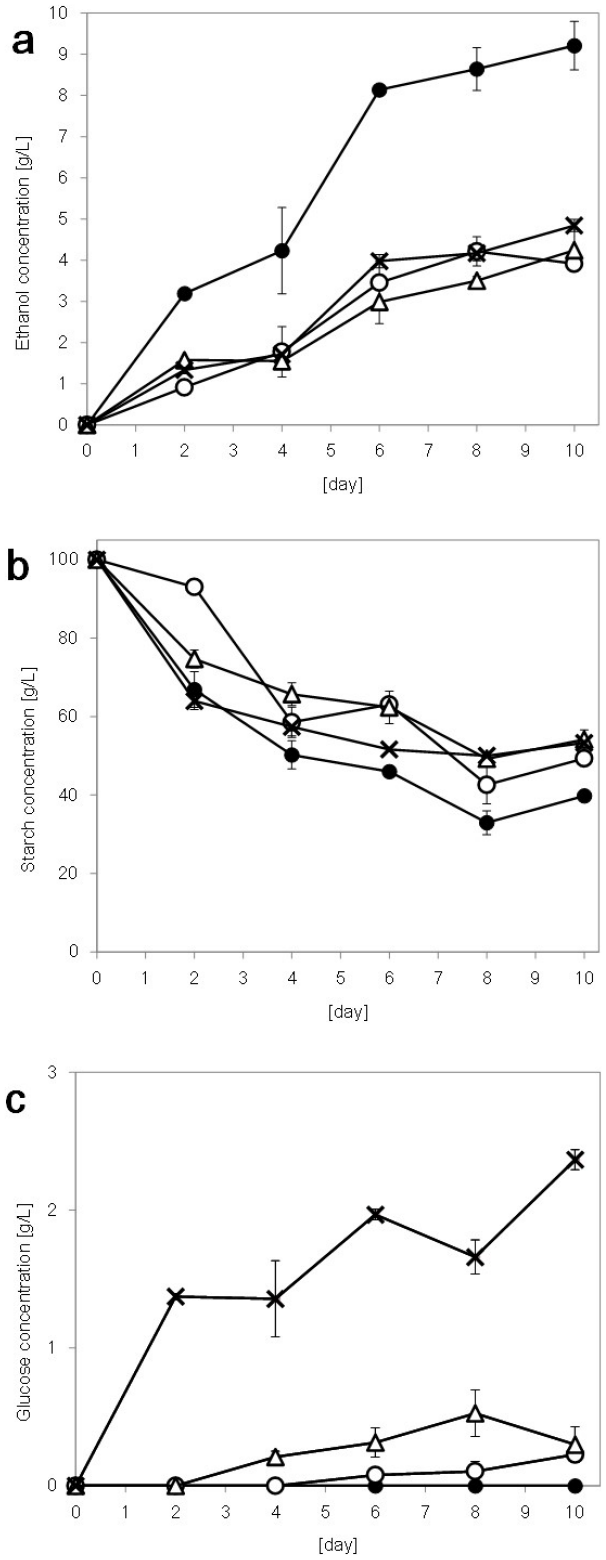
Changes in ethanol (a), starch (b) and glucose (c) concentrations of *S. shehatae* JCM 18690 (filled circles), ATY945 (open circles), ATY1112 (open triangles) and *S. shehatae* NBRC 1983 (crosses) from 10% starch liquid medium. Data are the means ± standard deviations (error bars) of three assays.

Ethanol production by the yeast strains was monitored over 10 days of cultivation in 10% starch liquid medium (Fig. 1a). After 7 days of cultivation, ethanol production by *S. shehatae* JCM 18690 reached approximately 8 g/L. In contrast, the ethanol productions by the other strains tested here were below 5 g/L even after 10 days of cultivation. These data suggested that *S. shehatae* JCM 18690 has unique characteristics which facilitate ethanol production from starch.

### Changes in starch and glucose during cultivation in starch medium

To gain insight into the ethanol-production ability of *S. shehatae* JCM 18690, changes in the concentrations of starch (Fig. 1b) and glucose (Fig. 1c) generated by amylases from starch were monitored in 10% starch liquid medium.

As shown in Figure 1b, *S. shehatae* JCM 18690 degraded starch faster than the other strains. However, after 10 days of cultivation, approximately 40% of the starch still remained in the medium. In the cases of the other strains, 50% of the starch was not utilized. These data implied that a high level of starch-degradation ability contributed to the ethanol production ability of *S. shehatae* JCM 18690. The comparisons of starch degradation will be described in detail in a later section. In addition, the disappearance of starch appeared to reach a plateau after 6 days of cultivation, and this trend was consistent with the curves of ethanol production. Since the ethanol production reached a maximum value at 6 days, it should be possible to shorten the fermentation period and make the process more time-efficient. However, as mentioned above, there was much unconsumed starch in the medium, and further work is needed to solve this problem.

To determine the behavior of the glucose released from the starch, changes in the glucose concentration during the 10-day cultivation were monitored (Fig. 1c). In cultivation broth containing *S. shehatae* JCM 18690, no glucose was detected during cultivation. These data strongly suggested that *S. shehatae* JCM 18690 ferments glucose simultaneously with the starch degradation. In contrast, in cultivation broth containing *S. shehatae* NBRC 1983, significant amounts of glucose remained in the broth, suggesting that the fermentation rate of *S. shehatae* NBRC 1983 was low. It is possible that the fermentation ability using glucose of *S. shehatae* JCM 18690 is higher than that using other strains and contributes to the ethanol production. Comparisons of the fermentation potential will be described in detail in a later section.

### Comparisons of α-amylase and glucoamylase activities

**Figure 2:**
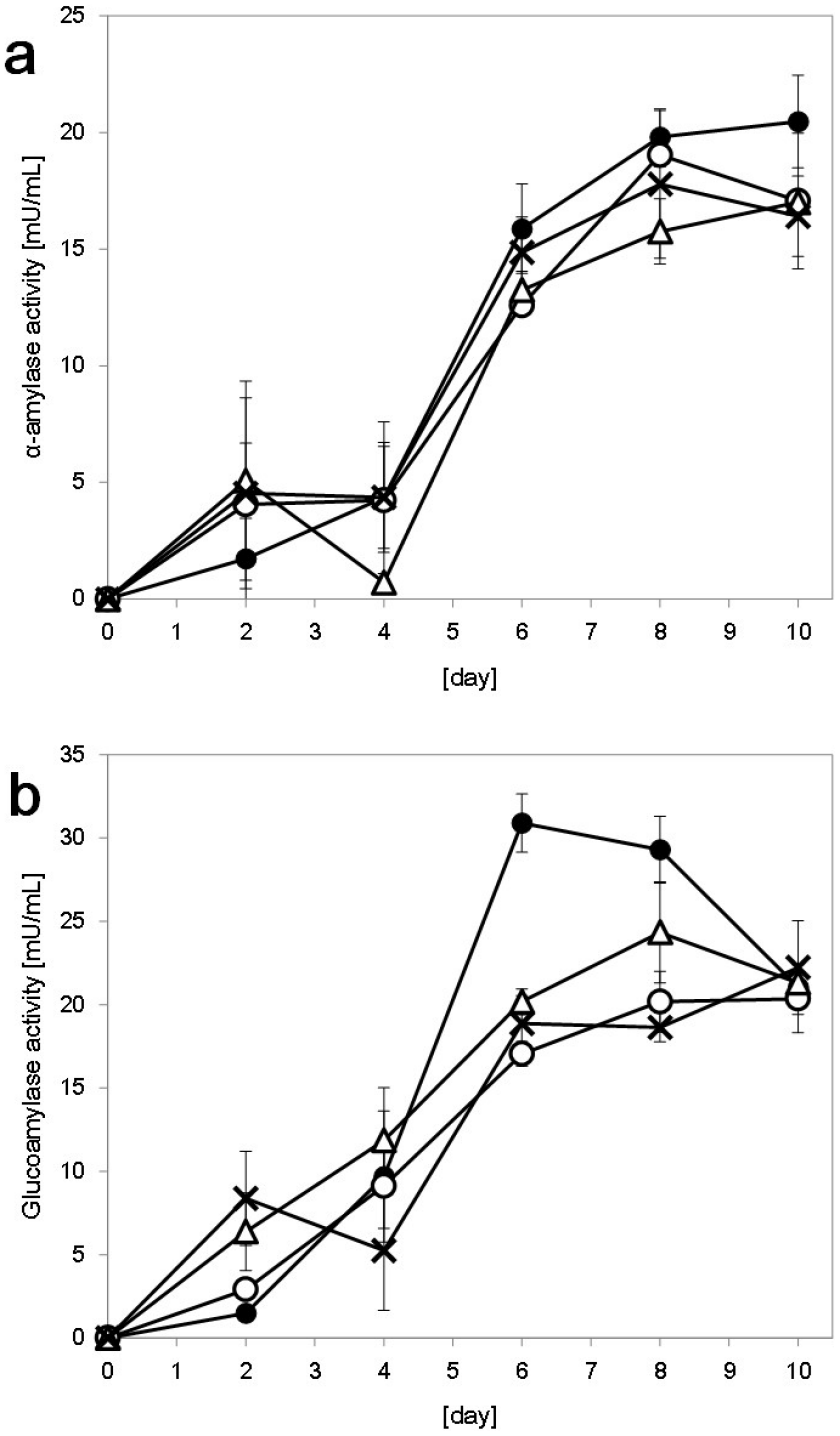
Changes in α-amylase (a) and glucoamylase (b) activity of *S. shehatae* JCM 18690 (filled circles), ATY945 (open circles), ATY1112 (open triangles) and *S. shehatae* NBRC 1983 (crosses). Data are the means ± standard deviations (error bars) of three assays.

Based on the data presented in Figure 1b, we hypothesized that the levels of starch degradation activity were correlated with the ethanol production of *S. shehatae* JCM 18690. Therefore, we examined the levels of α-amylase (Fig. 2a) and glucoamylase (Fig. 2b). In the α-amylase assay, we found no significant differences in α-amylase activity except for the samples collected after 10 days of cultivation. In contrast, as shown in Fig. 2b, the glucoamylase activity of *S. shehatae* JCM 18690 was significantly higher than those of the other strains. These data support the hypothesis that the high starch-degradation ability of *S. shehatae* JCM 18690 contributes to its ethanol-production ability.

### Comparisons of fermentation potentials

**Figure 3:**
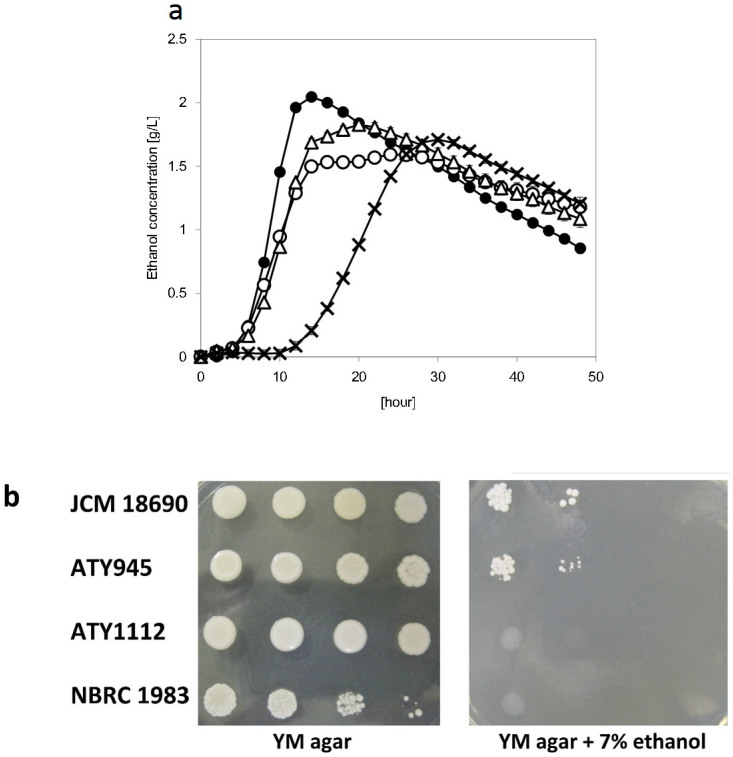
Fermentation potentials (a) and ethanol stress tolerance (b). Panel (a) shows the ethanol production of *S. shehatae* JCM 18690 (filled circles), ATY945 (open circles), ATY1112 (open triangles) and *S. shehatae* NBRC 1983 (crosses) from YM liquid medium containing 1% glucose. Data are the means ± standard deviations (error bars) of three assays. In panel (b), the tested strains were spotted onto YM agar medium or YM agar medium containing 7% ethanol in 10-fold serial dilutions from an initial OD_600_ value of 1.

Based on the data in Figure 3a, we speculated that the high level of fermentation potentials, which is defined as ethanol production ability from glucose, contributed to the ethanol production ability of *S. shehatae* JCM 18690. To determine the fermentation potentials, ethanol production from glucose was monitored using an automatic apparatus (Fig. 3a). As expected, the ethanol production rate of *S. shehatae* JCM 18690 was significantly higher than those of the other yeast strains. These data showed that the high fermentation potential of *S. shehatae* is correlated with its ability to produce ethanol from starch.

### Ethanol tolerance of the strains

We speculated that ethanol tolerance may contribute to the ethanol production ability of *S. shehatae* JCM 18690. To determine the level of ethanol tolerance, we performed a spot test on medium containing 7% ethanol. As shown in Figure 3b, *S. shehatae* JCM 18690 had relatively higher ethanol tolerance than *S. shehatae* NBRC 1983, ATY1112 and ATY945. These results indicate that ethanol tolerance is a required characteristic for an ethanol-producing strain.

## Discussion

CBP integrates enzyme production, saccharification and fermentation into a one-step process, and represents a promising strategy for the cost-effective production of ethanol from starchy biomass. To design commercial systems of ethanol production by CBP, it is necessary to develop customized microorganisms. Several previous attempts of ethanol production using various microorganisms are summarized in Table 1. *S. cerevisiae* strains have been genetically engineered to be capable of producing ethanol within a relatively short timeframe^17^,^18^. However, as described in the Introduction, the use of recombinant yeast strains increases production costs as well as biological risks^19^. Longer incubation times are generally needed for ethanol production using natural isolates of bacteria and fungus. Okamoto et al. reported that white rot fungus *Trametes hirsuta* was useful for the conversion of various biomasses to ethanol^20^. However, the ethanol conversion process proposed by Okamoto et al. included pre-cultivation for 1 week. This process is impractical because of the time inefficiency. In this study, we successfully identified *S. shehatae* JCM 18690 as the yeast strain showing the highest ethanol concentration, i.e., approximately 8 g/L within 1 week (Fig. 1a). To the best of our knowledge, *S. shehatae* JCM 18690 appears to be one of the most suitable naturally occurring strains for starch-based ethanol production yet discovered. Its rate of ethanol production (0.92 g/L/d after 10 days), i.e., productivity (g ethanol per liter of culture per day) is comparable to the best data reported in the literature: 0.9 g/L/d after 10 days from corn starch by *S. cerevisiae*^21^.

Among the many yeasts that have been studied for ethanol production, *S. erevisiae* remains one of the most promising, as shown in Table 1. Since *S. cerevisiae* lacks the starch-degrading ability, two strategies were attempted to express the amylase genes in the strain: the secretion of amylase into the medium and the anchoring of amylase to the cell surface^17^,^22^,^23^,^24^,^25^. The engineered amylolytic *S. cerevisiae* showed starch-hydrolyzing activities, but the process required a nutrient-rich medium (yeast extract and/or peptone and/or glucose)^18^,^23^,^26^. On the other hand, Liao et al. showed that the ethanol yield of the amylase-secreting *S. cerevisiae* achieved approximately three times higher than the anchored strain^5^. The authors attributed that the obtained difference to the limitation of the anchored amylase activity to the vicinity of the cell wall. In this study, the amylase assays were performed after filtering the culture medium with membrane filter twice to remove the cells. This indicates that the strain *S. shehatae* JCM 18690 secretes amylases into the medium, at least partially, and the secreted amylases contribute CBP production of ethanol from starch. In another interesting study, saccharolytic enzymes were co-expressed in *S. cerevisiae* cells to provide synergistic digestive action on starchy and lignocellulosic feedstock^27^. The authors showed that the strain can be used to hydrolyze starch and cellulose simultaneously. *S. cerevisiae* possesses traits, e.g. high ethanol-producing ability, high inhibitor tolerance and advantageous GRAS (generally regarded as safe) status and is still a candidate for starch conversion.

Kinetic analysis of the released glucose concentration shows that *S. shehatae* JCM 18690 was the only isolate that completed fermented glucose after 10 days (Fig. 1c). This behavior reveals that the stain is Crabtree-positive. It is considered that Crabtree-positive strains repress their own respiration in the presence of glucose, and start ethanol fermentation^28^,^29^. The other strains may be Crabtree-negative, with glucose remaining in the media. *S. shehatae* NBRC 1983 in particular left much more glucose than the others. In a previous study^16^, strain *S. shehatae* JCM 18690 (formally called ATY839) was identified as *S. shehatae*; however, it may belong to a new species having specific catabolic pathways different from that of the *S. shehatae* strain. Sequencing of the whole genome of this strain is underway.

As for the α-amylase activity, there were no significant differences between the strains (Fig. 2a). However, the glucoamylase activity of *S. shehatae* JCM 18690 was significantly higher; specifically, the activity at 6 days of the strain was 1.6-fold higher than that of the other strains (Fig. 2b). Soluble starch, which consists of α-1,4 linked glucose units, has been shown to be degraded to short polymer chains by α-amylase secreted from the yeast, while glucoamylase immediately saccharifies the polymers to produce glucose^1^,^30^. The glucoamylase-activity-increasing pattern and the ethanol concentration patterns of the strains were related to each other (Fig. 1a). These results indicate that the increase in the ethanol concentration of *S. shehatae* JCM 18690 was due to the increase in its glucoamylase activity.

Ethanol tolerance is an important characteristic of ethanol-producing yeast. Figure 3b shows the results of a growth test by dilution spot assays on YM agar medium containing 7% ethanol. There was no ethanol tolerance in ATY1112 and *S. shehatae* NBRC 1983, as manifested by the sluggish curves in Fig. 1a. It is suspected that these strains did not survive past the half-way point in the fermentation period. However, the viabilities of the strains were monitored until 10 days (data not shown). The relatively high ethanol tolerance of *S. shehatae* JCM 18690 could be the one of the factors responsible for the high ethanol production of the strain.

In the 1990s, Reddy et al. and Büttner et al. demonstrated the starch CBP conversion using the yeast strains of *Saccharomycopsis fibuligera* (formerly. *Endomycopsis fibuligera*) and *Blastobotrys adeninivorans* (formerly. *Arxula adeninivorans*), respectively^12^,^13^. The protocol proposed by Reddy et al. used rich medium containing 1.5% yeast extract and 1.5% peptone^12^. Although the ethanol concentration was impressive, it is uncertain whether the strain ferment from starch even if the medium which does not contain yeast extract and peptone was used. On the other hand, Büttner et al. showed that the all strains they tested produced 9–17 g/L ethanol^13^. Although these strains could show high conversion of starch, it was demonstrated at low (1 or 5%) starch loadings. Delayed conversion of starch (12 days) is most likely because the inhibitory effect of ethanol on yeast growth due to the low ethanol tolerance. Our strain has a high capacity for xylose fermentation, and thus is also advantageous for ethanol production from lignocellulosic materials^16^.

At least two previous studies have examined the direct fermentation of xylan to ethanol by *Scheffersomyces stipitis,* a species related to *Scheffersomyces shehatae*^31^,^32^. It has generally been recognized that the degradation of xylan is more complex than that of starch because it consists of β-1,4-linked xylose units with substituents such as acetyl, arabinosyl and glucuronosyl residues^33^. We did not perform assays of xylan degradation. However, we believe that various traits of *Scheffersomyces shehatae*, such as its high amylase activities, high glucose-fermentation ability and high ethanol tolerance, are unique and may allow for the development of starch utilization.

Our study strongly suggests the possibility of designing a starch-based ethanol production process through consolidated bioprocessing using natural yeasts including *S. shehatae* JCM 18690. The single-step conversion of starch to ethanol by natural yeasts represents a significant step toward practical bioethanol production by CBP.

## Methods

### Media

YM agar medium (Difco, Detroit, MI, USA) was used for the maintenance of yeast strains. An agar medium with 10% starch (0.17% yeast nitrogen without ammonium sulfate and amino acids (Difco), 0.5% ammonium sulfate, 10% soluble starch [Wako Pure Chemical, Tokyo, Japan], and 2% agar) was used for the primary screening of starch-assimilating ability. A 10% starch liquid medium (0.17% yeast nitrogen without ammonium sulfate and amino acids, 0.5% ammonium sulfate and 10% soluble starch) was used for the secondary screening and kinetic analysis of ethanol-producing ability. YM liquid medium (Difco) was used for the preculture and kinetic analysis of fermentation ability on glucose. The ethanol tolerance of the strains was determined based on the visual assessment of viability on YM agar medium containing 7% ethanol.

### Yeast strains

Yeast strains (530 strains) were isolated from different natural sources obtained from the Kyoto area in Japan (approximate total, 100 samples)^16^. As a control strain, the starch-fermenting and ethanol-producing yeast strain *Scheffersomyces shehatae* NBRC 1983 was obtained from the National Institute of Technology and Evaluation (NITE) Biological Resource Center.

### Determination of starch, ethanol and glucose concentrations

Starch, glucose and ethanol concentrations were determined using an HPLC apparatus (Shimadzu, Kyoto, Japan) equipped with an Aminex Fermentation Monitoring Column (Bio-Rad Laboratories, Hercules, CA, USA) and Micro-Guard Cation H Refill Cartridges with a Standard Cartridge Holder (Bio-Rad Laboratories). The detector was an RID 10A refractive index detector (Shimadzu). The column was kept at 60°C using a CTO 20A column oven (Shimadzu). Sulfuric acid solution (5 mM) was used as the mobile phase at a constant flow rate of 0.6 mL/min.

### Primary screening of yeast strains suitable for ethanol production by CBP

The starch-assimilating abilities of various yeast strains were evaluated as a primary screening method to identify yeast strains useful for ethanol production by CBP. The tested yeast strains were streaked onto 10% starch agar medium and cultivated at 25°C for 6 days. Cell growth was observed visually.

### Secondary screening of yeast strains suitable for ethanol production by CBP

As a secondary screening of yeast strains useful for ethanol production by CBP, the ethanol production of the yeast strains that had been selected in the primary screening was monitored. The 419 yeast strains were inoculated into 1 mL of 10% starch liquid medium in 1.5-mL microtubes and incubated at 25°C with or without shaking (150 rpm) for 6 days. The ethanol concentration in the supernatants after cultivation was analyzed as described above.

### Tentative identification of selected yeast strains

The yeast strains selected by the secondary screening were taxonomically identified by 26S rDNA sequencing. The partial 26S rDNA of the strain was amplified by PCR and directly sequenced based on a previously described method^34^. The homology of the sequence was determined by the BLAST system of the DNA Data Bank of Japan (DDBJ).

### Measurements of ethanol, glucose and starch during cultivation

To determine the ethanol production kinetics, changes in ethanol, glucose and starch were monitored. The yeast strains selected by the secondary screening were precultured overnight at 30°C with reciprocal shaking at 150 rpm. The preculture was washed with distilled water and suspended in 20 mL of 10% starch liquid medium in test tubes to a cell optical density at 600 nm (OD_600_) of 3, and incubated at 30°C in static culture for 10 days. Samplings were performed after 2, 4, 6, 8 and 10 days of cultivation. In this assay, *S. shehatae* NBRC 1983 was used as a control strain. All experiments were performed in triplicate.

### Amylase assays

The α-amylase and glucoamylase activity were measured with an α-amylase measurement kit and a saccharifying ability measurement kit (Kikkoman Co., Tokyo, Japan), respectively. The activities were determined according to the manufacturer's instructions by measuring the absorbance at 400 nm.

### Fermentation potentials of the yeast strains

To obtain insights into the ethanol production mechanisms, the fermentation potentials of the yeast strains were evaluated. In this study, we used the ethanol production rates from simple glucose medium (YM medium) as an index of fermentation potential. The yeast strains selected by the secondary screening and the control strain were precultured at 30°C, inoculated into 20 mL of YM liquid medium at a final OD_600_ of 0.2, and incubated at 30°C for 48 h with reciprocal shaking at 50 rpm. Fermentation was monitored every 2 h using a Fermograph II (Atto, Tokyo, Japan).

### Tolerance to ethanol stress

The yeast strains selected by the secondary screening and the control strain were precultured at 30°C. Cells were pelleted down by centrifugation, washed with distilled water and suspended in water to an OD_600_ of 1. The cell suspension was serially diluted in sterile water (dilution series: OD_600_ = 10^−1^, 10^−2^ and 10^−3^). Five microliters of each dilution were spotted onto YM agar medium or YM agar medium containing 7% ethanol, and the plate was incubated at 30°C for 2 days or 7 days, respectively.

## Figures and Tables

**Table 1:** Ethanol production through CBP

Substrate	Microorganisms	Ethanol yield [g/L]	Reference
Cassava pulp	*Clostridium thermocellum* and *Thermoanaerobacterium aotearoense*	8.8 after 5 d	35
Corn starch	*Saccharomyces cerevisiae* Mnuα1	9 after 10 d	21
Wheat bran	*Paecilomyces variotii*	1.2 after 13 d	36
Starch	*Trametes hirsuta*	9.1 after 96 h*	20
Rice straw	*Trametes hirsuta*	3 after 96 h*	20
Corn stover	*Clostridium phytofermentans*	2.8 after 10 d	37
Raw starch	*Saccharomyces cerevisiae* F2	2.6 after 240 h	26
Raw starch	*Saccharomyces cerevisiae* F6	2.1 after 240 h	26
Soluble starch	*Saccharomyces cerevisiae* SR93	14.3 after 140 h	38
Soluble starch	*S. shehatae* JCM 18690	9.2 after 10 d	This study

*One week of preculture was needed before the incubation.
